# *bla*_NDM-1_–positive *Klebsiella pneumoniae* from Environment, Vietnam

**DOI:** 10.3201/eid1808.111816

**Published:** 2012-08

**Authors:** Rie Isozumi, Kumiko Yoshimatsu, Tetsu Yamashiro, Futoshi Hasebe, Binh Minh Nguyen, Tuan Cuong Ngo, Shumpei P. Yasuda, Takaaki Koma, Kenta Shimizu, Jiro Arikawa

**Affiliations:** Hokkaido University, Sapporo, Japan (R. Isozumi, K. Yoshimatsu, S.P. Yasuda, T. Koma, K. Shimizu, J. Arikawa);; Nagasaki University, Nagasaki, Japan (T. Yamashiro, F. Hasebe);; and National Institute of Hygiene and Epidemiology, Hanoi, Vietnam (B.M.. Nguyen, T.C. Ngo)

**Keywords:** Klebsiella pneumoniae, bla_NDM-1_, rmtB, bacteria, antimicrobial resistance, carbapenems, sequence type, ST283, metallo-β-lactamase, beta-lactamase, environmental source, environment, *Suggested citation for this article*: Isozumi R, Yoshimatsu K, Yamashiro T, Hasebe H, Nguyen BM, Ngo TC, et al. *bla*_NDM-1_–positive *Klebsiella pneumoniae* from environment, Vietnam [letter]. Emerg Infect Dis [serial on the internet]. 2012 Aug [*date cited*]. http://dx.doi.org/10.3201/eid1808.111816

**To the Editor:** The *bla*_NDM-1_ gene, which produces the New Delhi metallo-β-lactamase (NDM-1) enzyme, confers resistance to the carbapenem class of antimicrobial drugs and can be transferred among different types of bacteria. NDM-1 was identified in 2008 in Sweden from a patient from India who had been hospitalized in New Delhi (*1*). Since that report, *bla*_NDM-1_–positive bacteria have been identified from patients in several countries; most of these patients had a direct link with the Indian subcontinent (*2*). The spread of *bla*_NDM-1_ among bacterial pathogens is of concern not only because of resistance to carbapenems but also because such pathogens typically are resistant to multiple antimicrobial drug classes, which leaves few treatment choices available (*3**–**5*). In 2011, spread of *bla*_NDM-1_–positive bacteria in an environmental setting in New Delhi was reported (*6*).

The possible appearance of bacteria harboring *bla*_NDM-1_ in Vietnam is of concern because cultural and economic links between Vietnam and India are strongly established, including extensive person-to-person exchanges that could enable easy exchange of pathogens. In addition, Vietnam faces a serious problem of antimicrobial drug resistance because drugs are freely available and used in an indiscriminate fashion. Thus, once *bla*_NDM-1_–positive bacteria colonize persons in Vietnam, they would be able to spread easily and pose a serious public health threat.

During September 2011, we collected paired swab samples (1 for PCR, 1 for culture) of seepage water from 20 sites (rivers, lakes, and water pools in streets) within a 10-km radius of central Hanoi, Vietnam. Samples were transported in Transystem (COPAN Italia S.p.A, Brescia, Italy) to preserve bacteria and DNA. The 20 PCR swab specimens were squeezed out into 0.5-mL volumes of sterile water and centrifuged at 3,000 × *g* for 30 seconds; 1 μL of the resulting suspension was then used as PCR template to detect *bla*_NDM-1_ as described (*7*). Two samples were positive for *bla*_NDM-1_; these 2 samples were collected from the same river (Kim Nguu River) but at sites 3 km apart.

To isolate and identify the phenotype and genotype of *bla*_NDM-1_–positive bacteria, we repeatedly spread the 20 culture swab specimens onto Muller-Hinton agar (Nissui, Tokyo, Japan) containing 100 mg/L vancomycin (Nakalai, Kyoto, Japan) plus 0.5 mg/L meropenem (LKT Laboratories, St. Paul, MN, USA) until single colonies were obtained. Each colony was then subcultured by plating onto MacConkey agar (Nihon Seiyaku, Tokyo, Japan) containing 0.5 mg/L meropenem to ensure culture purity; colonies were identified by using API 20E strips (bioMérieux, Basingstoke, UK). MICs of these isolates for 13 antimicrobial drugs were calculated by using Etest (bioMérieux), and susceptibility data were interpreted by using Clinical and Laboratory Standards Institute guidelines (www.clsi.org).

We harvested several species of bacteria from the 2 seepage samples positive for *bla*_NDM-1_: *Acinetobacter baumannii*, *Klebsiella pneumoniae*, *Pseudomonas aeruginosa*, *P. fluorescens/putida*, and *P. luteola*. These isolates were placed onto media containing 0.5 mg/L meropenem, and bacterial DNA was extracted and used for the template for PCR analysis to detect *bla*_NDM-1_ as described (*7*). *bla*_NDM-1_ was detected in 3 *K. pneumoniae* isolates from each of the 2 positive samples (6 isolates total); this result was confirmed by sequencing. All 6 isolates were highly resistant to all β-lactam antimicrobial drugs, including carbapenems (Table). To detect another β-lactamase, multiplex PCRs were carried out as described (*8*); genetic variants *bla*_TEM_, *bla*_SHV_, *bla*_OXA_, *bla*_CTX-M_, *bla*_IMP_, *bla*_VIM_, and *bla*_KPC_ were not detected in any of the isolates other than *K. pneumoniae*. All 6 *K. pneumoniae* isolates were positive for *bla*_TEM_ and *bla*_CTX-M_ variants by PCR; these variants were confirmed as *bla*_TEM-1_ and *bla*_CTX-M-3_ by sequencing.

**Table:** Resistance to 13 antimicrobial drugs of *bla*_NDM-1_–positive *Klebsiella pneumoniae* isolates from the Kim Nguu River, Hanoi, Vietnam*

Antimicrobial drug	MIC, mg/L	
	Site X	Site Y
Piperacillin/tazobactam	64–>256	64–>256
Cefotaxime	48–>256	96–128
Ceftazidime	>256	>256
Ceftriaxone	96–>256	128–>256
Meropenem	8–>32	12–>32
Doripenem	4–>32	8–>32
Imipenem	6–>32	>32
Fosfomycin	3–8	8
Gentamicin	>1,024	>1,024
Tobramycin	384–>1,024	256–384
Ciprofloxacin	0.064–1.5	0.064
Colistin	0.19–2	0.125–0.38
Tgecycline	1.5–3	0.5–1.5

*MICs were interpreted by using Clinical and Laboratory Standards Institute guidelines (www.clsi.org).

Aminoglycosides are often used in the management of severe infectious diseases caused by gram-negative pathogens. 16S rRNA methylases were found to confer high levels of resistance to aminoglycosides such as amikacin, tobramycin, and gentamicin. The 6 *K. pneumoniae* isolates we found were highly resistant to gentamicin (MIC >1,024 mg/L) and tobramycin (MIC 256–>1,024 mg/L) (Table). Therefore, we screened genetic elements of 16S rRNA methylases (*rmtB*, *rmtC,* and *armA*) by PCR and detected *rmtB* in all 6 isolates (*9*). Multilocus sequence typing was applied for these 6 isolates; all were identified as *K. pneumoniae* sequence type 283 (*10*), which had not been reported as harboring *bla*_NDM-1_. The azide-resistant *Escherichia coli* strain J53 has been used as recipient for conjugation assay, which had been reported previously (*6*), but we found no transconjugant strain with *bla*_NDM-1_ on MacConkey agar containing 100 mg/L sodium azide and 0.5 mg/L meropenem.

Our results show that *bla*_NDM-1_–positive *K. pneumoniae* sequence type 283 is present in the Kim Nguu River, which flows through the central part of Hanoi at 2 sites. The isolates we obtained were also positive for 2 other β-lactamases, *bla*_TEM-1_ and *bla*_CTX-M-3_, were highly resistant to aminoglycosides related to *rmtB*, and showed mild elevation of MIC against ciprofloxacin up to 1.5 mg/L. Wide-scale surveillance of environmental and clinical samples in Vietnam and establishment of a strategy to prevent further spread of *bla*_NDM-1_ are urgently needed.
